# The Effect of Static Compression via Vibration Foam Rolling on Eccentrically Damaged Muscle

**DOI:** 10.3390/ijerph19031823

**Published:** 2022-02-05

**Authors:** Masatoshi Nakamura, Kazuki Kasahara, Riku Yoshida, Kaoru Yahata, Shigeru Sato, Yuta Murakami, Kodai Aizawa, Andreas Konrad

**Affiliations:** 1 Department of Physical Therapy, Faculty of Rehabilitation, Niigata University of Health and Welfare, 1398 Shimamicho, Kitaku, Niigata 950-3198, Japan; rpa18029@nuhw.ac.jp (K.K.); rpa18121@nuhw.ac.jp (Y.M.); rpa18001@nuhw.ac.jp (K.A.); 2 Institute for Human Movement and Medical Sciences, Niigata University of Health and Welfare, 1398 Shimamicho, Kitaku, Niigata 950-3198, Japan; hpm21017@nuhw.ac.jp (R.Y.); hpm20011@nuhw.ac.jp (K.Y.); hpm19006@nuhw.ac.jp (S.S.); 3 Institute of Human Movement Science, Sport and Health, Graz University, Mozartgasse 14, 8010 Graz, Austria

**Keywords:** muscle strength, range of motion, countermovement jump height, pain pressure threshold, muscle soreness

## Abstract

Previous research has shown that vibration foam rolling (VFR) on damaged muscle can result in improvements in muscle soreness and range of motion (ROM). Furthermore, static compression via VFR (i.e., VFR without rolling) can increase the ROM and decrease the muscle stiffness of non-damaged muscle. Therefore, it is likely that static compression via VFR on eccentrically damaged muscle can mitigate muscle soreness and the decrease in ROM, and the decrease in muscle strength. The purpose of this study was to investigate the acute effects of a 90 s bout of VFR applied as a static compression on an eccentrically damaged quadriceps muscle, measuring ROM, muscle soreness, muscle strength, and jump performance. This study was a single-arm repeated measure design. Study participants were sedentary healthy male volunteers (n = 14, 20.4 ± 0.8 years) who had not performed habitual exercise activities or any regular resistance training for at least 6 months before the experiment. All participants performed a bout of eccentric exercise of the knee extensors with the dominant leg and then received a 90 s bout of static compression via VFR of the quadriceps 48 h after the eccentric exercise. The knee flexion ROM, muscle soreness at palpation, and countermovement jump height were measured before the eccentric exercise (baseline), before (pre-intervention) and after the VFR intervention (post-intervention), and 48 h after the eccentric exercise. The results showed that the static compression via VFR significantly (*p* < 0.05) improved the knee flexion ROM (6.5 ± 4.8%, d = 0.76), muscle soreness at palpation (−10.7 ± 8.6 mm, d = −0.68), and countermovement jump height (15.6 ± 16.0%, d = 0.49). Therefore, it can be concluded that static compression via VFR can improve muscle soreness and function.

## 1. Introduction

Vibration foam rolling (VFR) is a self-care technique involving the simultaneous use of foam rolling (FR) and vibration therapy, which shows the potential to have a favorable warm-up effect in increasing range of motion (ROM) [1] or performance [2] compared to conventional FR. In fact, recent studies have shown that VFR can increase range of motion (ROM) [1,3,4] and decrease muscle stiffness [3,5,6]. It has also been reported that VFR can increase muscle strength and jump performance [2,3], although the changes in these variables were not significant [5,6]. The additional vibration stimulus of VFR is likely the reason why VFR has been found to be more effective in increasing the ROM of a joint [1] and preparing for performance tasks [2], compared to normal FR. The possible superior effect of VFR over FR alone could be attributed to the greater changes in mechanoreceptors, e.g., Pacinian corpuscles caused by vibration stimulation [7].

Another interesting approach with FR is to perform a static compression on the target location without rolling. Wilke et al. (2018) showed that static compression at the trigger point using FR increases the pain pressure threshold (PPT) [8]. In addition, a study using VFR reported that static compression of the muscle belly and muscle–tendon junction increased ROM without decreasing muscle strength or jump performance [5]. Interestingly, this study also reported that static compression via VFR on the muscle belly was effective in reducing the shear elastic modulus. It is assumed that vibration stimulation induces greater changes in mechanoreceptors [7], promoting a decrease in shear elastic modulus. Thus, static compression via VFR could be used in sports in the future.

The importance of resistance training for sports and health promotion is well known, but intense training, and especially resistance training, including eccentric (ECC) contraction after a long time without sports or training, is known to cause delayed-onset muscle soreness (DOMS). Previous studies have revealed that repeated muscle contractions, especially ECC, could attenuate DOMS, the so-called “repeated-bout effect” [9,10]. However, since DOMS can influence athletic performance, reduce training quality, reduce adherence to resistance training, and result in a higher injury prevalence, it is necessary to control DOMS. Previous studies have shown that static stretching and FR can be effective for damaged muscle. Matsuo et al. (2015) reported that a 300 s static stretching intervention for the hamstring muscles could mitigate muscle soreness and the decrease in ROM, and increase passive stiffness [11]. In addition, Nakamura et al. (2020b) reported that a 90 s FR intervention for the quadriceps muscle could also mitigate muscle soreness and the decreases in ROM and muscle strength [12]. Moreover, Romero-Moraleda et al. (2019) compared the effect of FR and VFR on damaged muscles [13]. This previous study showed that both FR and VFR resulted in similar improvements in countermovement jump (CMJ) height, oxygen saturation, and PPT, but the VFR intervention resulted in greater improvements in pain and ROM than the FR intervention [13]. In addition, we compared the effects of VFR using different frequencies on damaged muscle and found that the improvements in ROM and muscle soreness were comparable [14]. Thus, the previous study concluded that it is not necessary to perform VFR at a high frequency to improve muscle soreness and function [14]. However, especially when applied to damaged muscles, VFR is accompanied by pain, which places a heavy burden on the participant. Therefore, if DOMS and loss of muscle function can be improved by static compression via VFR, it will be possible to establish a method that can easily improve ROM, muscle soreness, and muscle strength, with less burden on the participant. Therefore, the aim of this study was to investigate the effect of a static compression intervention via VFR on eccentrically damaged muscle. According to previous studies [5,8,13], we hypothesized that static compression on the muscle belly would improve ROM, muscle soreness, PPT, muscle strength, and jump performance.

## 2. Materials and Methods

### 2.1. Experimental Design

**Figure 1:**
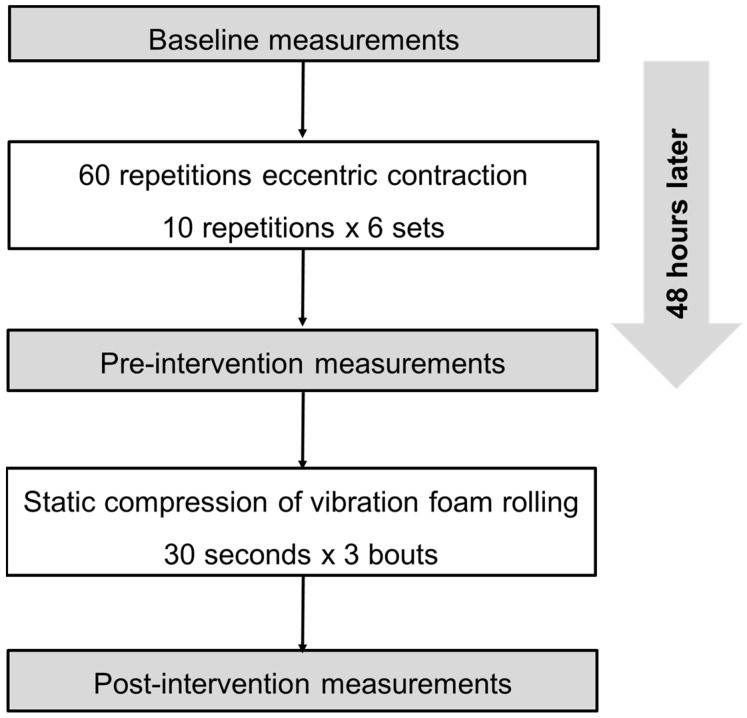
Experimental flow chart.

This study was a single-arm repeated measure design. We recruited all participants in our university from March to October 2021. In this period, we investigated the effect of static compression of VFR on DOMS. The outcome measurements consisted of knee flexion ROM; maximal voluntary isometric contraction (MVC-ISO) torque and maximal voluntary concentric contraction (MVC-CON) torque of the knee extensors; CMJ height; PPT; tissue hardness; muscle soreness at MVC-ISO and MVC-CON; and stretching before the maximal ECC task (baseline) and before (pre-intervention) and after the intervention (post-intervention) (Figure 1). Post-intervention measurements were performed immediately after the VFR intervention. All measurements were taken at the same time of the day for each participant. The participants were familiarized with all the measurements and ECC exercises before baseline measurement in the measurement leg (dominant leg). Our previous study already confirmed the high reliability of these outcome variables [15].

### 2.2. Participants

We investigated the static compression effect of VFR (i.e., without rolling) on eccentrically damaged muscle. Fourteen sedentary healthy young male volunteers participated in the study (age 20.4 ± 0.8 years; height 170.9 ± 6.8 cm; body mass 65.1 ± 9.3 kg). We included the sedentary healthy young males who had not performed habitual exercise activities. In particular, the regular resistance training, including ECC, could attenuate DOMS. Thus, we confirmed by asking that all participants had not been involved in any regular resistance training or flexibility training for at least 6 months before the measurements performed in this study. We excluded participants who had a history of neuromuscular disease or musculoskeletal injury on the lower extremity. All subjects were fully informed of the procedures and purpose of the study, and all gave written informed consent. The study complied with the requirements of the Declaration of Helsinki and was approved by the Ethics Committee of the Niigata University of Health and Welfare, Niigata, Japan. The sample size required for a one-way repeated analysis of variance (ANOVA) (effect size = 0.50, α error = 0.05, and power = 0.80) using G* power 3.1 software (Heinrich Heine University, Düsseldorf, Germany) was 14 participants.

### 2.3. MVC-ISO and MVC-CON

MVC-ISO torque of the knee extensor was measured using a dynamometer (Biodex System 3.0, Biodex Medical Systems Inc., Shirley, NY, USA) at 20° and 70° knee flexion angles. We instructed the participant to perform two maximal contractions of the knee extensors for 3 s at 20° and 70°, with a 60 s rest between trials. The average value for each angle was adopted for further analysis [15]. In addition, the MVC-CON torque of the knee extensor was measured for three continuous extensions (an angular velocity of 60°/s, and an ROM of 70° (20–90° knee angles)) [15]. The highest value among the three trials was adopted for further analysis. Verbal encouragement was provided during all the tests.

### 2.4. Knee Flexion ROM

Each participant was placed in a side-lying position on a massage bed, and the hip and knee of the non-dominant leg were flexed at 90° to prevent pelvis movement during the ROM measurements [12,14,15]. The investigator then brought the dominant leg to full knee flexion, with the hip joint in a neutral position. Finally, a goniometer was used to measure the knee flexion ROM three times, and the average value was used for further analysis.

### 2.5. Muscle Soreness

We measured the muscle soreness via a visual analog scale. This scale had a continuous line of 100 mm in length, with “not sore at all” on one side (0 mm) and “very, very sore” on the other side (100 mm). The magnitude of the knee extensor muscle soreness was assessed by muscle contraction (during MVC-ISO and MVC-CON measurement), stretching (during knee flexion ROM measurement), and palpation [10,12,15]. For muscle soreness during palpation, the participant was asked to lie supine on a massage bed. The investigator palpated the proximal, middle, and distal points of the vastus medialis, vastus lateralis, and rectus femoris [12,15,16]. The average value of the knee extensor palpation points was then used for further analysis. As for muscle soreness during stretching, the ROM measurement was performed three times, and the average value of muscle soreness was used for further analysis.

### 2.6. Pain Pressure Threshold 

In the supine position, we used an algometer (NEUTONE TAM-22 (BT10); TRY-ALL, Chiba, Japan) to measure PPT. The measurement position was defined as the midway point of the distance between the anterior superior iliac spine and the upper end of the patella of the dominant side for the rectus femoris muscle [15]. The investigator used the algometer to compress the soft tissue in the measurement area with continuously increasing pressure. The participant was instructed to press a trigger when minimal pain was experienced rather than just pressure. The value from the device at this time point was defined as the PPT value. PPT measurements were repeated three times at 30 s intervals, and the average value of three measurements was taken for further analysis [17,18].

### 2.7. Tissue Hardness

Tissue hardness was assessed via a portable tissue hardness meter (NEUTONE TDM-Z2; TRY-ALL Corp., Chiba, Japan). The participant’s measurement position and posture were the same as in the PPT measurement [15]. The participant was instructed to relax during the tissue hardness measurements. The tissue hardness measurements were repeated three times. We used the average value of three repetitions for further analysis.

### 2.8. Countermovement Jump Height

CMJ height was calculated using a jump mat system (Jump mat system; 4Assist, Tokyo, Japan). The participant started with the foot of the dominant leg on the mat, with their hands in front of their chest. The participant was then instructed to dip quickly (ECC phase) from this position, reach a self-selected depth, and jump as high as possible in the next concentric phase. The landing was performed on two feet. The knee of the uninvolved leg was held at approximately 90° of flexion [19]. After three familiarization repetitions, three sets of CMJs were performed and the jump height was measured. The maximum vertical jump height was used for further analysis [14,15].

### 2.9. Eccentric Exercise Task

The participant performed 60 (10 repetitions x six sets) unilateral maximal knee ECC extensions of the dominant leg via the isokinetic dynamometer [12,14,15]. The participant sat on the dynamometer chair at an 80° hip flexion angle. Moreover, the participant’s trunk, pelvis, and thigh were fixed strictly with adjustable Velcro straps. The participant was instructed to perform the maximal ECC contractions from a slightly flexed position (20°) to a flexed position (110°) at an angular velocity of 60°/s [12]. After each ECC contraction, the lever arm passively returned the knee joint to the starting position at 10°/s, which gave a 9 s rest between contractions. Each contraction was repeated 10 times, and a 100 s rest was given between sets for a total of six sets. The participant received strong verbal encouragement to generate maximum force during each ECC contraction.

### 2.10. Static Compression via Vibration Foam Rolling

A foam roller (Stretch Roll SR-002, Dream Factory, Umeda, Japan, dimensions: 150 × 320 mm; weight 870 g) was used for the VFR intervention [14]. Before the VFR intervention, a physical therapist instructed the participant on how to use the foam roller. In detail, the physical therapist explained where to place the leg on the foam roller, the amount of the vibration frequency, and the required compression. The VFR intervention was performed at 35 Hz, with 30 s bouts of VFR intervention and a 30 s rest between each set. The participant was instructed to remain in the plank position, with the foam roller at the middle position of the quadriceps of the dominant leg only. The participant was asked to compress as much body mass on the roller as was tolerable.

### 2.11. Statistical Analysis

SPSS (version 24.0; SPSS Japan Inc., Tokyo, Japan) was used for the statistical analysis. The data distribution was assessed using a Shapiro–Wilk test, and we confirmed that the data followed a normal distribution. One-way repeated measure ANOVA with Bonferroni post hoc test was used to determine the differences between the measurements taken at baseline, pre-intervention, and post-intervention. We also calculated the effect size (Cohen’s d) as the difference in the mean value divided by the pooled standard deviation (SD) between pre- and post-intervention in each group. A Cohen’s d value of 0.00–0.19 was considered as trivial, 0.20–0.49 as small, 0.50–0.79 as moderate, and ≥0.80 as large [20,21]. The difference was considered to be statistically significant at an alpha level of *p* < 0.05. Data are presented as mean ± SD.

## 3. Results

Table 1 lists the changes in knee flexion ROM, MVC-ISO torque, MVC-CON torque, and CMJ height at the baseline, and before and after static compression via VFR. One-way repeated measure ANOVA showed the significant main effects in these variables. Post hoc test showed that all the variables were found to be significantly reduced after the ECC exercise task, and the VFR without rolling significantly mitigated the knee flexion ROM (*p* < 0.01, change = 6.5 ± 4.8%, d = 0.76), MVC-CON torque (*p* = 0.021, change = 8.9 ± 8.7%, d = 0.21), and CMJ height (*p* < 0.01, change = 15.6 ± 16.0%, d = 0.49), but not the MVC-ISO torque (*p* = 0.26, change = 6.2 ± 9.8%, d = 0.14). However, the post-intervention knee flexion ROM, MVC-ISO torque, MVC-CON torque, and CMJ height values were significantly lower than the baseline values.

Table 2 shows the changes in PPT, tissue hardness, and muscle soreness at MVC-ISO, MVC-CON, stretching, and palpation, at baseline and before and after the static compression via VFR. One-way repeated measure ANOVA showed the significant main effects in these variables. PPT values at pre-intervention decreased significantly compared with baseline values. However, the values significantly improved after the VFR intervention compared to the baseline values (*p* < 0.01, change = 1.4 ± 0.5 kg, d = 2.17). Similarly, tissue hardness at pre-intervention increased significantly compared with baseline values. The values then significantly improved after the VFR intervention (*p* < 0.01, change = −10.7 ± 9.7%, d = −0.79) compared to the baseline values. For muscle soreness at MVC-ISO, MVC-CON, stretching, and palpation, the pre- and post-intervention values were significantly higher than the baseline values. All the variables improved significantly after the VFR intervention (muscle soreness at MVC-ISO: change = −14.0 ± 10.1 mm, d = −0.69, muscle soreness at MVC-CON: *p* < 0.01, change = −20.0 ± 18.8 mm, d = −0.82, muscle soreness at stretching: *p* < 0.01, change = −12.3 ± 8.9 mm, d = −0.59, muscle soreness at palpation: *p* < 0.01, change = −10.7 ± 8.6 mm, d = −0.68).

## 4. Discussion

In this study, we investigated the acute effect of static compression via VFR (i.e., VFR intervention without rolling) on eccentrically damaged muscle. The results showed that static compression via VFR could induce recovery from the decreases in knee flexion ROM, CMJ height, and increases in muscle soreness and tissue hardness. Interestingly, the effect of static compression via VFR is comparable to that of VFR with rolling [14]. However, static compression via VFR can be performed more easily and is less painful than VFR with rolling. Thus, static compression via VFR could be effective as a treatment for DOMS in athletes and older adults.

The results showed that static compression via VFR could significantly increase knee flexion ROM and decrease muscle soreness while stretching. The results also suggested that the decrease in muscle soreness while stretching can increase knee flexion ROM. In addition, a previous study showed that the decrease in ROM in eccentrically damaged muscle is related to increased tissue hardness [15]. The results of this study showed that tissue hardness is decreased after static compression via VFR. Therefore, the decrease in tissue hardness could contribute to the increase in knee flexion ROM, in addition to the decrease in muscle soreness while stretching. Interestingly, the changes in these variables were 6.5 ± 4.8% (d = 0.76) and −12.3 ± 8.9 mm (d = −0.59), and these changes were comparable with our previous study investigating the effect of VFR with rolling on damaged muscle (6.1 ± 4.4% and −13.6 ± 8.0 mm, respectively) [14]. Integrating these results, if the goal is to increase knee flexion ROM and decrease muscle soreness while stretching, it is not necessary to perform VFR with rolling. 

The results showed that static compression via VFR could improve MVC-CON torque significantly, but the effect size is small (d = 0.21, 8.9 ± 8.7%). However, muscle soreness at both MVC-ISO and MVC-COM recovered significantly after static compression via VFR. There is a possibility that the mitigation of muscle soreness can improve muscle strength, but the present study did not support this possibility. The discrepancy between the results of this study and the previous study could be related to vibration-induced muscle fatigue. Previous studies have suggested that vibration stimulation can cause post-activation performance enhancement by neural potentiation and can induce muscle fatigue [22,23]. Therefore, although the recovery in muscle soreness through static compression via VFR might have improved muscle strength, the vibration stimulation could have caused muscle fatigue, resulting in no significant change in muscle strength. However, static compression via VFR increased CMJ height significantly, which is consistent with our previous study [14]. This discrepancy might be because knee extension MVC-ISO torque and MVC-CON torque were measured using single-joint motion, whereas CMJ height was measured using multi-joint motion.

As shown in Table 2, PPT and muscle soreness were mitigated after static compression via VFR. Although the detailed mechanism of the decrease in muscle soreness is unclear, previous studies [7,24,25] have suggested that the proposed global pain modulatory response might be related to the gate control theory of pain, diffuse noxious inhibitory control, or parasympathetic nervous system alterations. In addition, VFR could induce selective activation through pressure and vibration and the large rapid contraction of muscle, thus improving the pain sensation [13]. Therefore, the analgesic effect of static compression via VFR might be caused by such mechanisms. Interestingly, the changes in PPT and muscle soreness after static compression via VFR were comparable with the changes after VFR with rolling [14]. Since static compression via VFR is easier and more comfortable than VFR with rolling, we propose the use of static compression via VFR as a new intervention method for eccentrically damaged muscle. However, future study is needed to investigate the effect of static compression via VFR on damaged muscle in athletes and the older population.

This study had several limitations. There was no control group (no VFR intervention group) in this study. However, the previous study [12] confirmed the high reliabilities for some outcome variables in eccentrically induced damaged muscles. Thus, there was a possibility that the static compression of VFR intervention could cause the recovery effect in this study. Moreover, the participants were sedentary young males and not athletes. The repeated resistance training, especially including ECC, could attenuate DOMS, the so-called “repeated-bout effect” [9,10]. Therefore, the loss of muscle function and muscle soreness in athletes might be less than DOMS in sedentary people, as seen in this study. Thus, the recovery effect of the static compression of VFR might occur, but it might be smaller than that of the present study. It is necessary to conduct similar studies with athletes in the future. In addition, it might be difficult for the older population to perform static compressions of VFR in the same position, such as plank training, as in this study. Therefore, it is necessary to study a method easily applied to the older population. However, the static compression of VFR used in this study could improve the loss of muscle function and muscle soreness more easily than the conventional FR and VFR intervention with movements. Therefore, it is expected to be applied to various age groups and athletes.

## 5. Conclusions

In conclusion, in this study, we investigated the acute effect of a 90 s static compression via VFR on eccentrically damaged muscle and showed that this intervention could improve knee flexion ROM, MVC-CON torque, CMJ height, tissue hardness, and muscle soreness. Therefore, it is concluded that static compression via VFR is an effective recovery method for eccentrically damaged muscles and is both easy and comfortable.

## Figures and Tables

**Table 1:** Changes (mean ± SD) in knee flexion ROM, maximal voluntary isometric contraction (MVC-ISO) torque of the knee extensors, maximal voluntary concentric contraction (MVC-CON) torque at 60°/s, and countermovement jump (CMJ) height, before the maximal eccentric contraction task (baseline), and pre- and post-static compression via VFR. The one-way repeated measure ANOVA results (*p*-value, F-value, and partial η^2^ [η_p_^2^]) are shown in the bottom row.

	Knee Flexion ROM (deg)	MVC-ISO Torque (Nm)	MVC-CON Torque (Nm)	CMJ Height (cm)
Baseline	135.5 ± 8.1	157.8 ± 32.1	168.1 ± 36.7	18.4 ± 3.2
Pre-intervention	118.2 ± 10.2 *	101.9 ± 34.8 *	103.7 ± 41.3 *	13.4 ± 3.8 *
Post-intervention	125.6 ± 9.2 *^, #^	106.6 ± 31.7 *	112.5 ± 42.7 *^, #^	15.0 ± 3.0 *^, #^
ANOVA results	*p* < 0.01, F = 46.3, η_p_^2^ = 0.78	*p* < 0.01, F = 27.1, η_p_^2^ = 0.68	*p* < 0.01, F = 19.5, η_p_^2^ = 0.62	*p* < 0.01, F = 23.8, η_p_^2^ = 0.65

*: A significant (*p* < 0.05) difference from the baseline value. ^#^: A significant (*p* < 0.05) difference from the pre-intervention value.

**Table 2:** Changes (mean ± SD) in pain pressure threshold (PPT), tissue hardness, muscle soreness at maximal voluntary isometric contraction (MVC-ISO), maximal voluntary concentric contraction (MVC-CON), stretching, and palpation, before the maximal eccentric contraction task (baseline), and pre- and post-static compression via VFR. The one-way repeated measure ANOVA results (*p*-value, F-value, and partial η^2^ [η_p_^2^]) are shown in the bottom row.

	PPT (kg)	Tissue Hardness (N)	Muscle Soreness at MVC-ISO (mm)	Muscle Soreness at MVC-CON (mm)	Muscle Soreness at Stretching (mm)	Muscle Soreness at Palpation (mm)
Baseline	2.2 ± 0.8	18.2 ± 3.6	7.7 ± 6.1	7.0 ± 7.7	2.1 ± 4.6	12.9 ± 10.7
Pre-intervention	0.9 ± 0.5 *	20.8 ± 2.5 *	46.3 ± 20.2 *	49.0 ± 22.9 *	42.6 ± 21.9 *	44.3 ± 17.3 *
Post-intervention	2.3 ± 0.7 ^#^	18.6 ± 3.0 ^#^	32.3 ± 20.2 *^, #^	31.2 ± 20.2 *^, #^	30.3 ± 19.4 *^, #^	33.6 ± 14.2 *^, #^
ANOVA results	*p* < 0.01, F = 41.0, η_p_^2^ = 0.76	*p* < 0.01, F = 10.2, η_p_^2^ = 0.44	*p* < 0.01, F = 29.4, η_p_^2^ = 0.69	*p* < 0.01, F = 29.1, η_p_^2^ = 0.71	*p* < 0.01, F = 35.1, η_p_^2^ = 0.73	*p* < 0.01, F = 33.0, η_p_^2^ = 0.72

*: A significant (*p* < 0.05) difference from the baseline value. ^#^: A significant (*p* < 0.05) difference from the pre-intervention value.

## Data Availability

All data supporting the conclusions of this study will be fully provided upon request by the authors.
